# Evaluation of Macular Retinal Vessels and Histological Changes in Two Cases of COVID-19

**DOI:** 10.3390/biomedicines9111546

**Published:** 2021-10-26

**Authors:** Maria Hernandez, Jorge González-Zamora, Sergio Recalde, Maite Moreno-Orduña, Valentina Bilbao-Malavé, Manuel Saenz de Viteri, Manuel F. Landecho, Patricia Fernandez-Robredo, Alfredo García-Layana

**Affiliations:** 1 Retinal Pathologies and New Therapies Group, Experimental Ophthalmology Laboratory, Department of Ophthalmology, Clinica Universidad de Navarra, 31008 Pamplona, Spain; jgzamora@unav.es (J.G.-Z.); maimoreno@unav.es (M.M.-O.); vbilbao@unav.es (V.B.-M.); msaenzdevit@unav.es (M.S.d.V.); pfrobredo@unav.es (P.F.-R.); aglayana@unav.es (A.G.-L.); 2 Navarra Institute for Health Research—IdiSNA, 31008 Pamplona, Spain; 3 Red Temática de Investigación Cooperativa Sanitaria en Enfermedades Oculares (Oftared), 31008 Pamplona, Spain; 4 COVID-19 Unit, Clínica Universidad de Navarra, 31008 Pamplona, Spain; mflandecho@unav.es; 5 Department of Internal Medicine, Clinica Universidad de Navarra, 31008 Pamplona, Spain

**Keywords:** SARS-CoV-2, COVID-19, macula, retina, vasculature, ACE2, tortuosity, microglia

## Abstract

The purpose of this study was to assess vascular and histological alterations in two COVID-19 and three control post-mortem retinas. The macular areas of flat-mounted samples were processed for immunofluorescence. Lectin and collagen IV positive vessels were captured under confocal microscopy, and endothelium loss and tortuosity were analyzed. Expression of ACE2 (angiotensin-converting enzyme 2) (the receptor for SARS-CoV-2), Iba1 (ionized calcium-binding adaptor molecule 1) and GFAP (glial fibrillary acidic protein) were quantified in retinal sections. The number of lectin vessels in COVID-19 retinas decreased by 27% compared to the control (*p* < 0.01) and the tortuosity increased in COVID-19 retinas (7.3 ± 0.2) vs. control retinas (6.8 ± 0.07) (*p* < 0.05). Immunofluorescence analysis revealed an increase in ACE2 (2.3 ± 1.3 vs. 1.0 ± 0.1; *p* < 0.0001) and Iba1 expression (3.06 ± 0.6 vs. 1.0 ± 0.1; *p* < 0.01) in COVID-19 sections whereas no changes in GFAP were observed. Analysis of the COVID-19 macular retinal tissue suggested that endothelial cells are a preferential target of SARS-CoV-2 with subsequent changes through their ACE2 receptor expression and morphology. Thus, microglial activation was hyperactive when facing an ensuing immunological challenge after SARS-CoV-2 infection.

## 1. Introduction

In December 2019, the first cluster of cases was reported in Hubei Province, China; since then, coronavirus disease 2019 (COVID-19) has spread worldwide, infecting more than 214 million people and causing more than 4.47 million deaths as of 27 August 2021 [1]. Patients with COVID-19 show a wide range of systemic manifestations, including neurological and ocular involvement [2,3]. However, which pathogenic mechanisms lead to the alterations observed in affected patients remain unknown. Microvascular alterations, combined with cytokine overproduction, have profound implications for the development of multisystem organ failure and have been proposed to be triggered by widespread endothelial cell damage [4,5].

To enter human cells, the virus uses a spike protein, angiotensin-converting enzyme 2 (ACE2) receptor, which has been detected in the retina [6]. Viral particles have also been detected in the human retina [7,8]; moreover, patients with COVID-19 can experience ocular and neurological signs and symptoms [9,10,11,12,13]. These data, combined with the findings of several microvascular-related abnormalities, make the retina a potential biomarker for studying systemic vascular disease, owing to the ease of measuring minor changes in microvascular perfusion by optical coherence tomography angiography (OCTA) [10,14,15].

While OCTA can be used to quantify blood flow in the capillary plexi, superficial capillary plexus, deep capillary plexus, and choriocapillaris, it provides no information on the etiology of these changes in vascular perfusion. There are currently limited histological retinal descriptions of the abnormalities that occur during infection and even fewer that focus on the microvasculature. Araujo-Silva et al. observed viral particles within endothelial cells close to the capillary flame and cells of the inner and outer nuclear layers [7], while Jidigam et al. described decreased vascular density and increased inflammation and gliosis [16].

More research is needed to understand these microvasculature changes. We describe the macular alterations occurring in the entire retinal vasculature plexus and histological evidence, including glial and microglial responses, in post-mortem retinas of patients who died from COVID-19.

## 2. Materials and Methods

### 2.1. Human Donor Eyes

Five human donor eyes (*n* = 2 for COVID-19 and *n* = 3 for control donors) were used in this study (Figure S1, Table S1). Mean COVID-19 donor age was 81.5 years and 90.3 years for controls. Donors were obtained from the Department of Pathology, Anatomy and Physiology of the School of Medicine, University of Navarra and Clínica Universidad de Navarra. All donors provided informed consent in accordance with the Declaration of Helsinki and local ethical committee.

Tissue was processed within 2–48 h from death. An experimental overview is shown in Figure S1. COVID-19 samples were confirmed positive for antibodies against SARS-CoV-2 (SARS-CoV-2-specific IgG and IgM antibodies (S-RBD)) by Microbiology Department, Clínica Universidad de Navarra. Control samples were serologic negative for IgG and IgM.

### 2.2. Tissue Processing

The eyes were fixed in 4% paraformaldehyde diluted in phosphate buffer (PB) for 3 h and 2% paraformaldehyde for 6 days at 4 °C. Then the eyes were cryopreserved in 15% sucrose 24 h and 30% sucrose until use. Retinas were removed from optic cup, washed in phosphate buffer saline (PBS) and flat-mounted.

### 2.3. Immunofluorescence in Flat-Mounted Eyes and Retinal Sections and Conventional Hematoxylin-Eosin Staining in Retinal Cross-Sections

Retinal flat-mounts were incubated in blocking buffer containing (3% Triton X-100, 0.5% Tween 20, 2% sodium azide, and 1% fetal bovine serum (FBS) in PBS) for 5 h at 4 °C. Then, they were subjected to biotinylated isolectin (1:240, L8262 Sigma–Aldrich, Saint Louis, MO, USA) and anti-collagen IV (1:500, 1340-01, Southern Biotech, Birmingham, AL, USA) for 3 days at room temperature (RT). Samples were washed in PBS and incubated in Alexa Fluor streptavidin 488 (1:250; S32354; Life Technologies, Carlsbad, CA, USA) and donkey anti-goat Alexa Fluor 594 (1:250; A11058, Thermo Fisher Scientific, Waltham, MA, USA) for 3 h. Flat-mounts were mounted with PBS-glicerol (1:1).

After analyzing and capturing retinal flat-mount images, the retinas were embedded in OCT (Optimal compound tissue) and stored at −80 °C. Fourteen-micron frozen sections from areas adjacent to the macula were obtained containing the macular as well as the peripheral retinal region using a cryostat (Microm HM550; Thermo Fisher Scientific, Waltham, MA, USA). Sections were mounted onto glass slides and stored at −20 °C in a refrigerator until use. Three to four retinal sections per group were chosen, blocked with blocking buffer and incubated in target retrieval solution (Dako, Santa Clara, CA, USA) at 90 °C for 10 min. Collagen IV and lectin stained samples were subject to the following antibodies: mouse anti-glial fibrillary acidic protein (GFAP) (1:100; 3670S; Cell Signalling, Danvers; MA, USA), rabbit anti-ionized calcium-binding adaptor molecule 1 (Iba1), (1:100; CP290A; Biocare Medical, Concord, CA, USA) and rabbit anti-ACE2 (1:50; ab108252; Abcam, Cambridge, MA USA), and incubated overnight at 4 °C. After PBS washing, donkey anti-rabbit Alexa Fluor 647 (1:250; A31573; Invitrogen, Carlsbad, CA, USA) and donkey anti-mouse Alexa Fluor 647 (1:250; A31571; Invitrogen Carlsbad, CA, USA) were added and incubated for 1 h at RT. 4′,6-diamidino-2-phenylindole (DAPI) (Sigma-Aldrich, St. Louis, MO, USA) was used to stain the nuclei.

Conventional hematoxylin–eosin (H&E) staining for morphological observation of the retinal layers was performed in three sections per eye.

### 2.4. Confocal and Brightfield Image Capture and Analysis

All retina imaging was performed using a confocal microscope (LSM800; Zeiss, Oberkochen, Germany). For overview, Z-scanned pictures of retinal flat-mounts with lectin and collagen IV labelling were captured using a 10×/0.75 NA objective. Five sample areas of 14.7 mm^2^ per donor around the macula were captured and analyzed with ImageJ software (Figure S1). The number of lectin and collagen IV vessels was calculated using a custom Fiji/ImageJ [17] plugin that automatically detected vessel intersections from the 3D segmentation mask of the complete retinal vasculature (Figure S1). The endothelium loss rate was calculated as number of lectin vessels/number of collagen IV vessels. The same program was further customized to calculate simple tortuosity between branching points by calculating the average ratio between each individual vessel length (arc length) and the shortest distance between its opposite ends (chord length) [18]. This image analysis software was developed by the Imaging Platform of the Center for Applied Medical Research (CIMA).

Twenty cross retinal sections from 5 donors were used to measure staining intensities for ACE2, Iba1 and GFAP (ImageJ). The results were normalized to the unlabeled areas background staining of as presented by percentage.

Three images from each HE section were captured using a brightfield microscope (AxioImager, Zeiss, Oberkochen, Germany) to assess retinal morphology structure.

### 2.5. Statistical Analysis

Data are presented as mean ± SEM. Statistical analysis was performed using GraphPad Prism 8.0 (GraphPad Software, San Diego, CA, USA). Data were compared between COVID-19 and control eyes, by a paired Student t-test. The minimum level of significant difference was defined as *p* < 0.05.

## 3. Results

### 3.1. Endothelium Loss in COVID-19 Retinas

**Figure 1:**
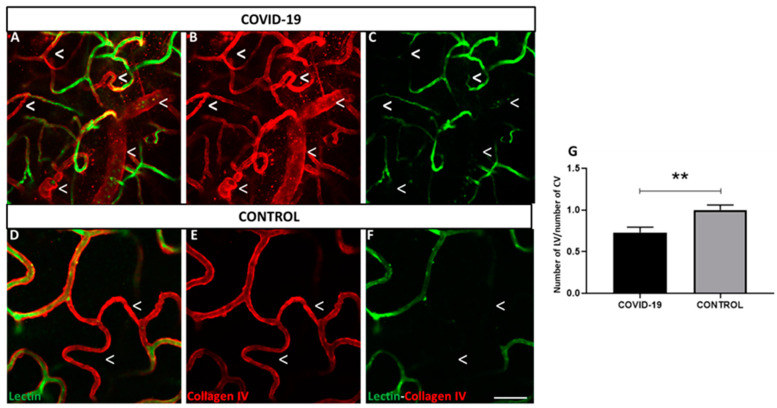
Retinal vessels labeled with lectin vs. collagen IV positive vessels. (**A**–**F**) Endothelial cells in retinal flat-mounts are labelled with lectin (green) and basement membrane is stained with collagen IV (red) in COVID-19 (**A**–**C**) and control retinas (**D**–**F**). (**G**) Percentage of number of lectin vessels (LV) vs. number of collagen IV vessels (CV). Expression of lectin decreased in endothelial cells in both groups; however, COVID-19 retinas showed less expression than controls. ** *p* < 0.01. Scale bar: 100 µm. Arrowheads indicate missing lectin endothelium areas.

The numbers of blood vessels labeled with lectin (endothelial cells) and collagen IV (basal membrane) in the total vascular layers in the human retina (Figure 1A–F) were quantified. Superficial, intermediate, and deep vascular layers were skeletonized (Figure S2) and quantified around the macula. The number of lectin vessels in the retinas of patients with COVID-19 was decreased by 27% compared to that in the control (*p* < 0.01) (Figure 1G, in which the basement membrane is stained red (collagen IV) and endothelial cells in green (lectin)). Despite of the clearly observed endothelium loss in COVID-19 vs. control flat-mounted retinas, no morphological alterations were observed in HE stained cross retinal sections (Figure S1E,F).

### 3.2. Tortuosity Increased in COVID-19 Retinal Vessels

**Figure 2:**
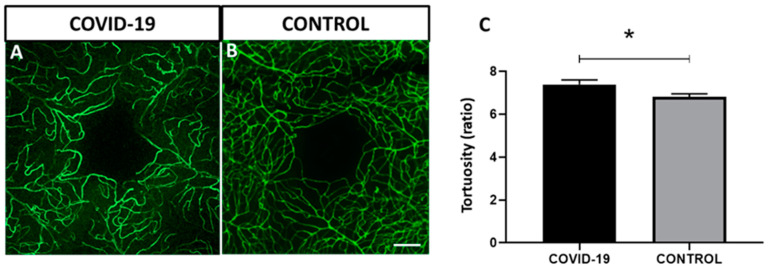
Vessel tortuosity in flat-mounted retinas in COVID-19 and control labelled with lectin (green). (**A**–**C**) Quantitative analysis in the macular area. Tortuosity is significantly increased in COVID-19 samples compared to controls. Error bars indicate S.E.M. * *p* < 0.05. Scale bar: 100 µm.

Analysis showed significantly increased tortuosity in the retinal vessels of the retinas in the patients with COVID-19 (7.3 ± 0.2) vs. control retinas (6.8 ± 0.07) (*p* < 0.05) in the macular area (Figure 2).

### 3.3. Pattern Localization and Quantification of ACE2 in Retinal Vessels

**Figure 3:**
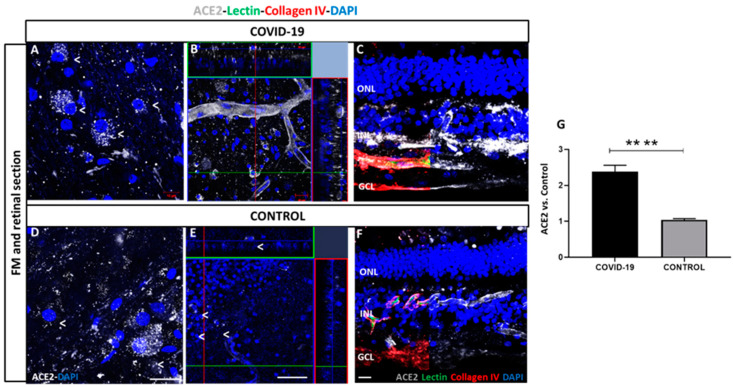
Angiotensin-converting enzyme 2 (ACE2) expression in COVID-19 (**A**–**C**) and control retinal vessels (**D**–**F**). ACE2 expression (white) observed in retinal ganglion cells (RGCs) in superficial vasculature in COVID-19 (**A**) and control (**D**) retinas. ACE2 is observed in cells in inner nuclear layer (INL) and ganglion cell layer (GCL) and endothelial cells in vessels of COVID-19 (**B**) and controls (**E**). ACE2 localization in Optimal compound tissue (OCT)-embedded retinas cross-sections previously labelled with lectin (green) and collagen IV (red) in COVID-19 (**C**) and controls (**F**). B and E represent an orthogonal projection of retinal vessels. 4′,6-diamidino-2-phenylindole (DAPI) (blue) label nuclei. (**G**) Quantification of immunofluorescence intensity in percentage of COVID-19 retinas vs. controls. **** *p* < 0.0001. Scale bar: 100 µm. Abbreviations: GCL (ganglion cell layer), INL (inner nuclear layer), ONL (outer nuclear layer).

ACE2 was expressed in capillaries throughout the retina, with the highest expression observed in retinal ganglion cells (RGCs) and vascular endothelial cells in vessels located in the RGCs of patients with COVID-19 (Figure 3A–C) and control retinas (Figure 3D–F). Flat-mounted (A,B) and cross retinal sections (C) of samples from the patients with COVID-19 showed strong staining in both types of cells compared to the control (D–F). ACE2 expression was quantified by measuring the relative fluorescence intensity, which clearly showed a significant ACE2 signal increase in COVID-19 retinas compared to controls (2.3 ± 1.3 vs. 1.0 ± 0.1) (Figure 3G, *p* < 0.0001).

**Figure 4:**
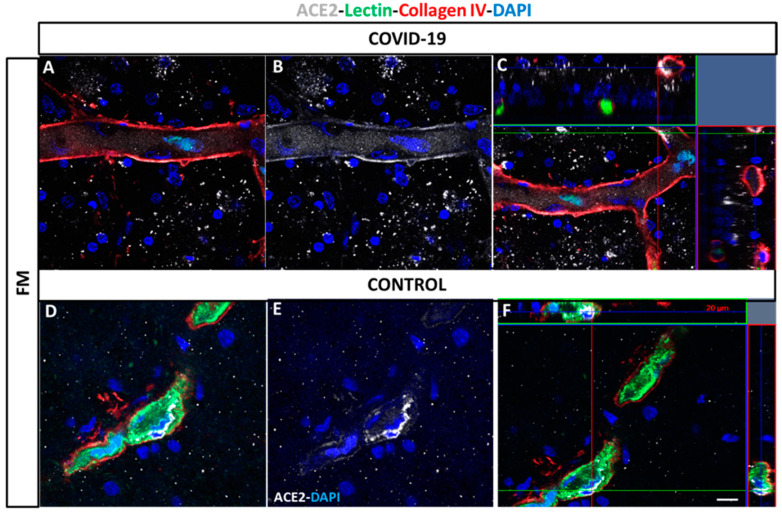
Localization of ACE2 in vessels in post-mortem flat-mounted COVID-19 (**A**–**C**) and control retinas (**D**–**F**). Samples label with lectin (green), collagen IV (red) and ACE2 white). ACE2 expression is analyzed in superficial and deep vasculature. ACE2: Angiotensin-converting enzyme 2, FM: flat-mounted. DAPI (blue) label nuclei. Scale bar: 200 µm.

At higher magnification, retinal vessels located in the superficial vasculature (Figure 4E) showed ACE2 positive cells in both COVID-19 (Figure 4A–C) and control retinas (Figure 4D–F).

### 3.4. Iba1 and GFAP Expression in COVID-19 Human Retinas

**Figure 5:**
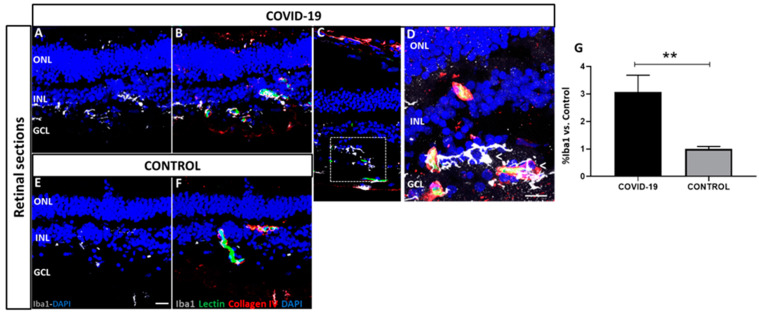
Localization and quantification of ionized calcium-binding adaptor molecule 1 (Iba1) (white) in COVID-19 (**A**–**D**) and control retinal cross-sections (**E**,**F**) previously labelled with lectin (green) and collagen IV (red). In contrast to controls (**E**,**F**), COVID-19 retinas (**A**–**D**) exhibited very strong Iba1 staining. Boxed region in (**C**) is shown at higher magnification in (**D**). DAPI (blue) label nuclei. (**G**) Percentage of Iba1 signal intensity from COVID-19 retinas vs. controls. ** *p* < 0.01. Scale bar: 100 µm. Arrows indicate areas of microglial activation. Abbreviations: GCL (ganglion cell layer), INL (inner nuclear layer), ONL (outer nuclear layer).

The expressions of Iba1 and GFAP throughout the retina in COVID-19 and control post-mortem samples were determined. Activated microglia (Iba1) were expressed in the inner nuclear layer (INL) and ganglion cell layer (GCL) (Figure 5). Notably, COVID-19 samples (Figure 5A–D) showed a three-fold increase in the immunoreactive profile of Iba1 (3.06 ± 0.6) compared to that in the controls (1.0 ± 0.1) (Figure 5E,F) (*p* < 0.01).

**Figure 6:**
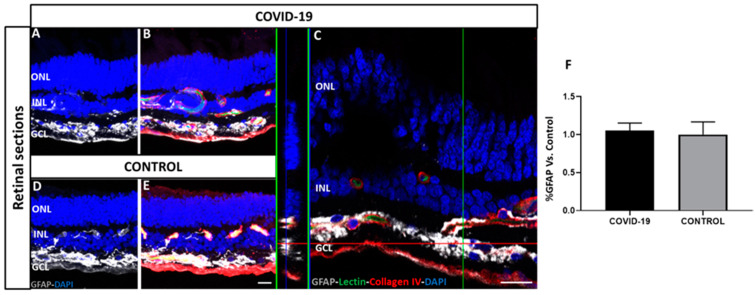
Localization and quantification of glial fibrillary acidic protein (GFAP) staining (white) in COVID-19 (**A**–**C**) and control retinal cross-sections (**D**,**E**) previously labelled with lectin (green) and collagen IV (red) antibodies. There were no differences between COVID-19 (**A**–**C**) and control retinas (**D**,**E**). Image C is a magnification of GFAP staining in GCL. DAPI (blue) labels nuclei. (**F**) Quantification of immunofluorescence intensity in percentage of COVID-19 retinas vs. controls shows no significant differences. Scale bar: 100 µm. Abbreviations: GCL (ganglion cell layer), INL (inner nuclear layer), ONL (outer nuclear layer).

Astrogliosis was observed in the retinal sections on GFAP immunostaining. Some GFAP-positive cells were found to extend their end-feet processes in the GCL surrounding blood vessels (Figure 6). However, no differences were found between COVID-19 and control retinas, and the quantification of signal intensity was similar in COVID-19 (1.05 ± 0.1) and controls (1.0 ± 0.3).

## 4. Discussion

This study documented vasculature alterations in the human retinal plexus and an increase in ACE2 expression, microglial activation, and astrogliosis in retinal layers after SARS-CoV-2 infection. One of the main results was the absence of endothelial markers in deep and superficial retinal plexus in controls that was more evident in retinas from patients with SARS-CoV-2 infection. This might indicate that the endothelium was severely damaged or even completely absent. Accordingly, increasing evidence suggests that a dysfunctional endothelium may be the main pathogenic mechanism of the prothrombotic state in COVID-19 [16,19,20,21]. Moreover, the detection of retinal vascular lectin loss is a feature of the aged human central nervous system and aged retinas with the previously described appearance of acellular capillary remnants in the periphery of human and rat retinas [22,23].

We also observed a significant increase in capillary tortuosity in COVID-19 retinas. Other authors indicated that microangiopathy might be secondary to COVID-19 or incidental, suggesting that the virus itself or the systemic treatments used might have triggered microangiopathy in patients with systemic vascular disease [16,24]. Vascular tortuosity at later stages of retinal vascular aging has been reported [23,25] in other diseases, such as diabetic retinopathy [26], familial retinal arteriolar tortuosity (fRAT) [27], chronic anemia [28], and facioscapulohumeral muscular dystrophy [29]. Despite the endothelial alterations found in flat-mounted retinas, morphologic analysis in retinal cross-sections did not show the hallmark changes from chronic diseases, such as microaneurysms, between COVID-19 cases and controls.

Apart from the morphological vascular alterations observed in the COVID-19-positive donors, we report the widespread expression of ACE2 in the retina. It is remarkable that retinal vasculature and RGCs expressed ACE2, with a particularly high density in COVID-19 retinas. As the signal was observed at the external part of the vessel, these cells probably corresponded to pericytes, consistent with reports in COVID-19 brain samples [30]. The potential impairment of these structures could be the neural substrates for the clinical manifestations of COVID-19 syndrome, as well as those described in the brain [30]. Recent histopathological studies from patients who died from severe COVID-19 indicate the presence of endothelial inflammation [31]; moreover, neuroinflammation during COVID-19 could be partially explained by changes in ACE2 expression at the blood–brain barrier of the vast brain network of capillaries, which could affect the integrity of endothelial tight junctions, thereby allowing passage of cytokines and inflammatory cells.

Furthermore, in the present study, we found evidence for increased microglial cells in the retinas of patients with COVID-19, which appear to show characteristic hallmarks of microglial hypertrophy, as reported previously [16,32]. Microglial cells changed with an increase in ramified morphology, and most migrated to the retinal vessels in the GCL and nerve fiber layer. Recent studies have shown that dystrophy is a disease associated with microglial morphology [32] and brain alterations in COVID-19 [33].

Astrocytes are key regulators of homoeostasis and respond to stimuli through the upregulation of GFAP and astroglial hypertrophy [34]. In our study, astrogliosis was evident in human retinas in both groups, with no difference between them. However, astrogliosis occurs in a variety of pre-existing medical conditions and has been proposed to exert protective effects against oxidative stress [35,36,37].

In particular, astrocytes in the aged human retina have been described to undergo morphological changes, including hypertrophy and increased density of intermediate filaments, displaying increased GFAP immunoreactivity [38,39]. Therefore, SARS-CoV-2 infection was not the direct cause of astrogliosis in the present study. However, a recent study observed an increase in GFAP immunoreactivity near the ONH regions in some cases of COVID-19 patients, but not near the middle retinal regions [16].

Overall, our findings are consistent with a growing body of evidence suggesting that endothelial cells are a preferential target of SARS-CoV-2 [40]. SARS-CoV-2 can infect endothelial cells using the ACE2 receptor, with subsequent endotheliitis and endothelial cell apoptosis [40]. The disruption of vascular integrity due to direct viral infection and immune-mediated inflammation leads to the exposure of the thrombogenic basal lamina and activation of the clotting cascade [19].

Amongst the limitations of the study, the main one could be the low number of samples analyzed to reach statistically significant conclusions. However, we consider that the magnitude of the changes found and their statistical significance, despite the small sample size, support the need to take them into consideration. Another limitation to be highlighted is that the changes we found seem relatively nonspecific. It can be argued that may be due to coexisting systemic disease or simply age dependent degenerations; however, controls were older than the COVID-19 donor. In addition, the lack of specific criteria owing to the nature of the samples means that we do not know the concomitant diseases of any donor included, both controls and COVID-19. Therefore, more studies with a greater number of retinal samples are needed. Finally, the severity of the viral infection is unknown, and it was not possible to the use clinical history of donors in our study.

Future work to confirm the results obtained is needed. In this sense, we are planning to also study other molecules involved in the virus entry to different cells, such as Neuropilin 1. In addition, of special importance is the role of pericytes in endothelium alterations, thus we will analyze pericyte involvement by immunofluorescence. Finally, we plan to assess gene and protein changes in future collected samples.

## Data Availability

All data are available within the manuscript and upon request to corresponding author.

## Supplementary Materials

The following are available online at https://www.mdpi.com/article/10.3390/biomedicines9111546/s1, Figure S1: Summary with a table human donors information and experimental design, Figure S2: Images of the macular area studied in superficial, intermediate, and deep vascular layers of COVID-19 (A,F,B,G) and control (C,H,D,I,E,J) donors.
