# Supplementary material for: Evaluation of Macular Retinal Vessels and Histological Changes in Two Cases of COVID-19
Source: Biomedicines. 2021 Oct 26;9(11):1546. doi: 10.3390/biomedicines9111546 (PMC8615149; doi:10.3390/biomedicines9111546)
Supplement: Supplementary file 1 [file biomedicines-09-01546-s001.zip › biomedicines-1400764-supplementary.pdf]

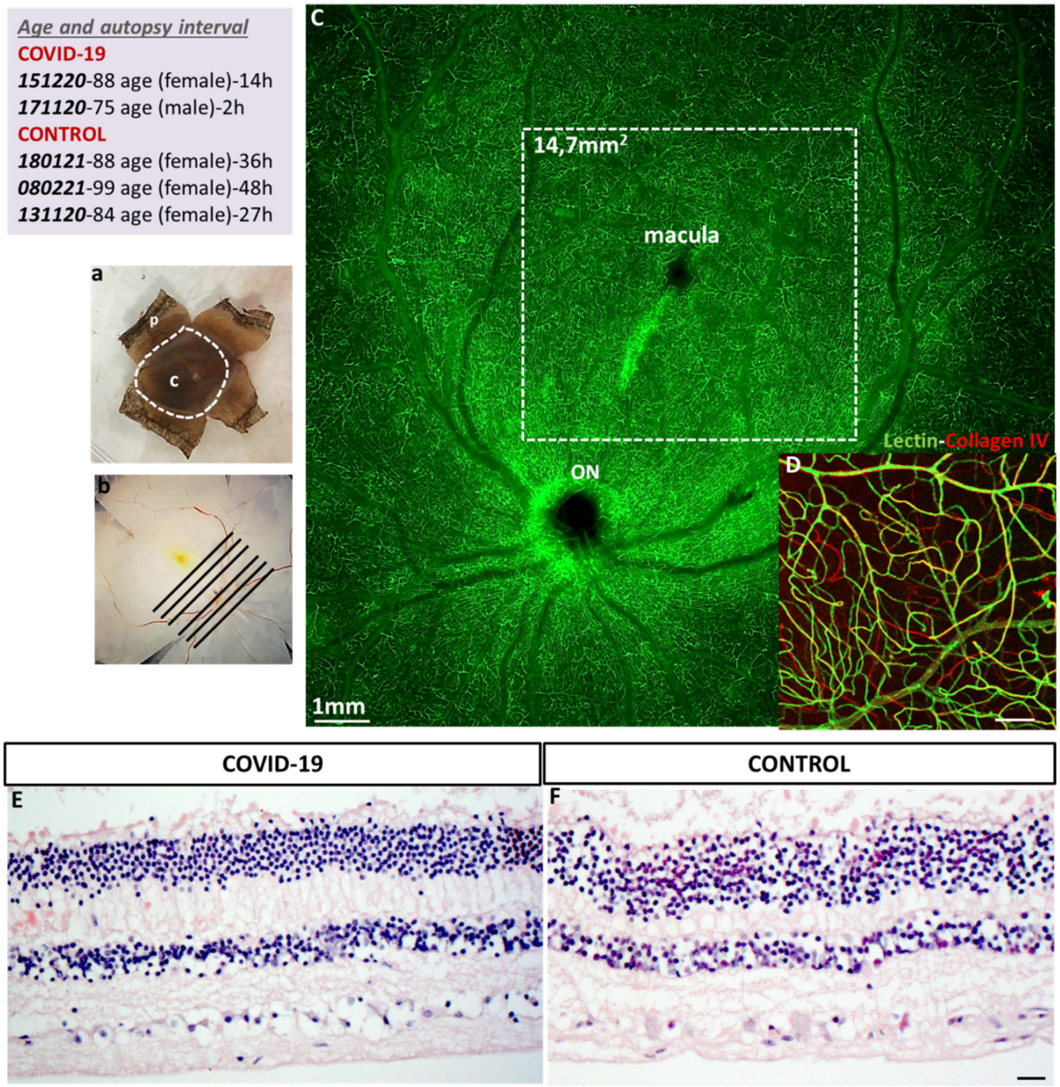

Figure S1. Summary with human donors information (table), image of human RPE-flat-mounted retina (a), scheme of human retina used for perform immunofluorescence in retinal sections near to macula (b), COVID-19 retinal flatmount labeled with lectin (C) and collagen IV (C and D) in the area studied (14.7 mm^2). HE images of retina in COVID (E) and control (F) retinas in optic nerve area. Scale bar (C): 1 mm, (D): 500 µm, (E and F): 200 µm. Abbreviations: p (periphery), c (central), ON (optic nerve).

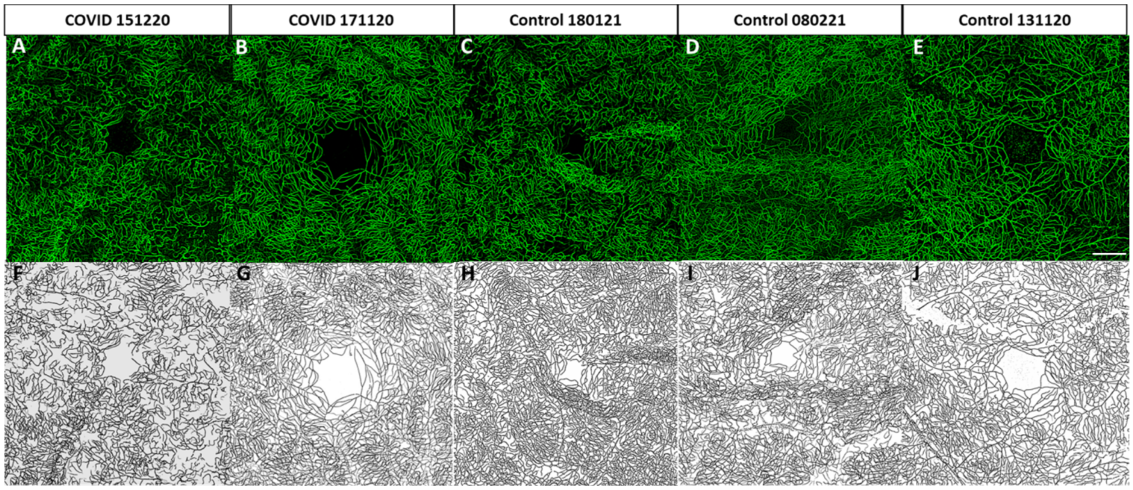

Figure S2. Images of the macular area studied in superficial, intermediate, and deep vascular layers of COVID-19 (A, F, B, G) and control (C, H, D, I, E, J) donors. (A-E) Flatmounts of macular samples labeled with lectin. (F-J) Skeletonized images of the lectin images for vascularl analysis.
